# Not in Their Right Mind? Right-Wing Extremism Is Not a Mental Illness, but Still a Challenge for Psychiatry

**DOI:** 10.3389/fsoc.2022.830966

**Published:** 2022-05-11

**Authors:** Frank Schumann, Peter Brook, Martin Heinze

**Affiliations:** Department of Psychiatry and Psychotherapy, Brandenburg Medical School, Immanuel Klinik Rüdersdorf, Rüdersdorf, Germany

**Keywords:** psychiatry, right-wing extremism, radicalization, psychiatrization, right-wing populism, mental health, prevention

## Abstract

Most research in psychiatry on extremism focuses on the question whether there is a connection between extremism and psychiatric diagnoses. In addition, practitioners are increasingly asked to take part in programs aimed at preventing and countering violent extremism by assessing risk for radicalization. However, an issue that remains largely unaddressed is that the rise of the far right in many countries during the last years poses a challenge for psychiatric services as working with right-wing patients can be a source of conflict for practitioners and patients alike. In this article, we assert that the narrow conceptual scope on psychological vulnerabilities and the practical focus on risk assessment contribute to processes of psychiatrization and limit the scope of research on right-wing extremism in psychiatry. By giving a brief overview of social research into right-wing extremism, the article argues that right wing beliefs should not be conceptualized as an expression of psychological vulnerabilities but rather as attempts to deal with conflict-laden social reality. Thus, a shift of perspective in psychiatric research on extremism is needed. On a conceptional level, the scope needs to be broadened to grasp the interplay of individual and social factors in radicalization with sufficient complexity. On a practical level, it is necessary to further investigate challenges for practitioners and institutions working with right-wing extremist patients.

## Introduction

In the last 10 years, right-wing populism^1^ has established itself as a stable force within the political spectrum of many countries, in some even as part of the government (Mudde, 2019). This led to intense debates not only in the public sphere, but also in research on the causes and consequences of right-wing extremism. After radicalization prevention had focused mainly on Islamism in the years after the September 11 attacks, the recent developments brought right-wing extremism back into focus.

As with many sectors of public life, the popularity of the far right, and the accompanying public focus on it, also affect psychiatric services. As public health care institutions, psychiatric institutions must offer services to right-wing extremists just as to other patients. This results in various challenges. For example, the presence of right-wing extremists can be a burden for other patients or staff, especially if they have experienced discrimination related to racism and right-wing extremism. On the other hand, and unlike other health care institutions, psychiatric clinics and services are increasingly confronted with a particular set of questions: Are right wing extremist orientations and violence connected to psychiatric diagnoses? What role should psychiatry play in prevention of radicalization and violence?

The assumption of a connection between psychopathology and extremist violence has a long history (Gilman and Thomas, 2016) and is found particularly in media and public debate (DeFoster and Swalve, 2018). In psychiatry and criminology, the notion of a psychopathology of extremists has been discussed controversially since the 1970's (Cooper, 1978; Tanay, 1987; Victoroff, 2005). Lack of empirical evidence was a source of repeated criticism over the years and led most researchers in the field of radicalization to reject the assumption of mental illness having a causal influence on radicalization processes. In recent years, however, psychiatric institutions have increasingly been included in programs aimed at preventing and countering violent extremism (P/CVE), most prominently in the US and the UK. This resulted in a certain revival of research on mental illness and extremism as evidenced by a growing corpus of empirical studies.

Although research into how extremism challenges psychiatric and psychotherapeutic institutions has become highly relevant in recent times, current approaches suffer conceptually from a one-sided focus on mental health problems. This conceptual reductionism is accompanied by a prevention perspective that combines public health and security agendas and thus drives a psychiatrization of a phenomenon that should rather be understood as a complex interplay of individual, social and societal factors. The article argues for a research perspective that can integrate social as well as societal context and is able to address practical challenges of public health care institutions in working with extremist patients. This is done by (1) giving a concise summary of the conceptual and practical shortcomings in psychiatric research on extremism and (2) contrasting psychiatric approaches with current social research on right-wing extremism. The shift in research perspective encouraged by the article comprises both violent as well as non-violent forms of extremism.

## Right-Wing Extremism and Psychiatry

### The Conceptual Perspective: Mental Health as a Risk Factor for Radicalization

For a long time, the psychiatric literature put forward the thesis of a psychopathology of extremists mostly based on conceptual considerations (Cooper, 1978; Tanay, 1987). However, during the last 10 years, an increasing number of studies were published that empirically investigate the presumed connection of extremism and mental illnesses (Gill and Corner, 2017). These studies report elevated prevalence rates for psychiatric diagnoses among extremists, particularly for depression (Bhui et al., 2014; Bhui, 2016; Campelo et al., 2018a; Rousseau et al., 2019; Morris and Meloy, 2020) but also for schizophrenia (Weenink, 2019), and personality disorders (Coid et al., 2016). Especially “lone wolf”-terrorists are more likely to fulfill the criteria for a psychiatric diagnosis (Gruenewald et al., 2013; Corner and Gill, 2015; Corner et al., 2016). Most studies follow a tendency noticeable in radicalization studies in general, namely, to focus primarily on Islamism, whereas right-wing extremism tends to play a minor role (Bjørgo and Aasland, 2019). Isolated studies, however, can be found claiming that right-wing violent offenders are more likely to have had traumatic experiences during childhood (Baron, 1997; Simi et al., 2016).

Although studies seem to point toward a connection of psychiatric diagnoses and radicalization, findings are inconsistent and do not allow for a clear conclusion. Meta-studies report considerable variation in prevalence rates between individual studies (Trimbur et al., 2021) and note that many studies have a weak diagnostic basis (Corner et al., 2021; Gill et al., 2021). The heterogenous findings do not come as a surprise, since radicalization is usually seen as a complex process with a wide range of pathways (Borum, 2012). As such, many factors do play a role in radicalization processes, for example, the presence of social adversities, availability of radical ideology, and proximity to radical political groups. In addition, radicalization is a highly dynamic process that encompasses personality changes (Bjørgo, 2011) and represents an independent source of psychological stress that must be dealt with (Koehler, 2020). Studies on the correlation of psychiatric diagnoses and extremism can neither account for the complexity nor for the dynamism of radicalization processes. It thus remains unclear, how elevated prevalence rates for psychiatric diagnoses among extremist populations are to be interpreted. Based on the available data, a causal connection of mental illness and radicalization cannot be established.

Nevertheless, psychiatric literature maintains that psychic vulnerabilities can help to explain extremism (Corner et al., 2021; Gill et al., 2021), if social and societal aspects are factored in (McCauley and Moskalenko, 2008; Simi et al., 2016; Decety et al., 2018; de Ridder et al., 2019; Gill et al., 2021; Harpviken, 2021). This is usually done by situating individual risk factors within a multilevel model that includes the social micro level (family, friends), meso level (communities, social class), and macro level (societal and political developments) (Doosje et al., 2016; Eisenman and Flavahan, 2017; Campelo et al., 2018b), often mirroring the ecological model for violence prevention by the World Health Organization (WHO, 2004).

Although, at first glance, paying closer attention to social context seems to increase the explanatory power of the model, central problems remain. Firstly, the model does not clarify how individual and social factors interact in radicalization processes (Smith et al., 2020). Instead, it is implied that individual, social, and societal risk factors just add up to an overall radicalization risk, neglecting the dynamism of radicalization processes and leaving open why, under otherwise similar conditions, some individuals develop extremist orientations and others do not. Secondly, adding social context does not solve the initial problem plaguing the explanatory model, namely, that there is little evidence for a causal connection between psychiatric diagnoses and extremism.

### The Practical Perspective: Mental Health Practices in the Context of Counterterrorism

In 2015, the United Kingdom Government revised the Prevent Strategy Policy that now requires psychiatrists among other professions to identify and report people at risk of being drawn into terrorism (HM Government., 2015; Weine et al., 2017; Chivers, 2018). Community-based programs aiming at preventing and countering violent extremism have also been launched in the United States (Ellis and Abdi, 2017). In the European Union, the Radicalization Awareness Network (RAN) encourages mental health practitioners to assess risks for radicalization (Al-Attar, 2019; RAN Practitioners., 2021a,b).

The prevention approach is often laid out within a public health framework (Bhui et al., 2012; McGilloway et al., 2015; Alcalá et al., 2017; Bhui and Jones, 2017; Bhui, 2018; Aggarwal, 2019; Weine and Kansal, 2019). Following Caplan (1964), these approaches usually make a distinction between primary, secondary, and tertiary prevention (Weine and Kansal, 2019). While primary prevention aims at anticipating radicalization processes before they occur, for example, by working with communities to increase social cohesion and access to social services (Ellis and Abdi, 2017), secondary prevention aims at identifying and intervening in radicalization processes at an early stage, and tertiary prevention seeks to rehabilitate extremists. Psychiatrists are usually asked to work in all three areas as part of a multidisciplinary team that also involves community work (Weine et al., 2017). Thus, the prevention approach contributes to an expansion of psychiatric tasks and structures.

However, the prevention approach goes beyond the public health field and is part of national security programs, some of which have already been mentioned. Within these programs, psychiatric structures are assigned the task of assessing risks for violent radicalization of patients as part of an early warning system and thereby contributing to preventing terrorist attacks (Eisenman and Flavahan, 2017; Weine et al., 2017). In some states, as Denmark for example, institutional structures have been created to coordinate collaboration between psychiatry, intelligence, police, and social work (Freestone, 2017; Sestoft et al., 2017). As such, psychiatry becomes part of a national security agenda. This point has been hotly debated among psychiatrists, especially in the UK. While some argue that psychiatrists are responsible to protect society from “violence resulting from mental illness” (Hurlow et al., 2016, p. 162) and therefore should cooperate with security agencies to prevent radicalization, many are skeptical about the scientific foundations of risk assessment procedures (Bhui, 2016; Royal College of Psychiatrists., 2016; Khoshnood, 2017) and point toward ethical issues such as negative stereotyping of Muslim communities and breaching medical confidentiality (Middleton, 2016; Summerfield, 2016).

Based on the dual perspective of public health and security agendas, practical recommendations for psychiatrists focus primarily on the question of what specific roles psychiatrists should play in P/CVE (Al-Attar, 2019; Dom et al., 2020) and how they can contribute to risk assessment (Eisenman and Flavahan, 2017; Logan and Lloyd, 2019; Bhui et al., 2020; Logan and Sellers, 2021). Several tools now exist to assist psychiatrists in risk assessment, such as Trap-18 (Meloy, 2018) and the VERA-2R (Pressman et al., 2016). The focus on safety issues and the tendency to view Muslim communities and psychiatric patients as “dangerous people” (McSherry and Patrick, 2011) has been the subject of repeated criticism (Coppock and MacGovern, 2014; Open Society Justice Initiative., 2016; Rizq, 2017; Abbas, 2019).

## The Role of Society: Right-Wing Extremism as Reaction to Social Conflict

It is widely assumed in radicalization literature that grievances within the social lifeworld of individuals work as a push factor in radicalization (Borum, 2012; Hafez and Mullins, 2015). Despite acknowledging that social factors play a role in radicalization, most studies on extremism in psychiatry still focus mainly on individual risk factors such as psychological vulnerabilities and do not go into much detail how these vulnerabilities interact with social and societal factors. In contrast, explanations developed in social research give a more nuanced account of societal developments leading to right-wing support. Although explanations differ, most of them agree on a crucial point: right-wing orientations need to be explained as a reaction to a social reality that is perceived to be in crisis.

One of the oldest explanations for right-wing extremism, the theory of the authoritarian personality developed by Adorno et al. (2019) during World War II, views right-wing extremism as an expression of a personality structure that formed in reaction to feelings of powerlessness caused by strict and punishing parents. If similar feelings of powerlessness are reexperienced later in life, for example during personal or social crises, authoritarian personalities are likely to turn toward right-wing political groups. While the original concept of authoritarianism was complemented by a social theory developed by Adorno, later social psychological reformulations, such as Right-Wing Authoritarianism (Altemeyer, 1981), pay less attention to societal conditions of authoritarianism. However, newer research again emphasizes social roots of right-wing extremist orientations by combining the concept with a theory of perceived threat (Onraet et al., 2013).

Recent social research on the popularity of the far right builds less on theories of authoritarianism and instead describes the turn to radical and extreme right-wing positions within the conceptual framework of status threat. It is an ongoing debate whether it is predominantly economic or cultural change that threatens social status. Socioeconomic explanations (Manow, 2018; Rodrik, 2018) highlight the role of economic insecurity following the transformation of economy and labor market due to globalization, while sociocultural approaches argue that a change of conventional values and norms is responsible for feelings of threat (de Wilde et al., 2019; Inglehard and Norris, 2019). Despite the differences, both explanations assume that a threat to social status drives right-wing support. Research on supporters of the German right-wing populist party Alternative für Deutschland (AfD) found that it is a combination of both socioeconomic and sociocultural threat that predicts support for the right-wing party (Lengfeld and Dilger, 2018).

Although less popular in recent years, another explanation for radicalization can be found in approaches that focus on anomie. These emphasize the dissolution of social cohesion and security following societal processes of individualization as an important factor for the development of right-wing extremist orientations (Anhut and Heitmeyer, 2009). Evidence for the anomie-theoretical explanation can also be found in the electorate of far-right parties such as the AfD. The impression of being increasingly socially isolated is widespread among AfD supporters (Müller-Hilmer and Gagné, 2018).

## Discussion

Even though studies report higher prevalence rates of psychiatric diagnoses among extremists, there is no clear evidence for a causal influence of mental health issues on extremism. Rather, as social research shows, right-wing extremist orientations form through a complex interaction of individual and social factors and can be understood as an attempt to come to terms with a social reality that is perceived to be in crisis. Psychiatric research into right-wing extremism should, therefore, be able take the interplay of individual and social factors within the biography of extremists into account. Against this background, current research on right-wing extremism in psychiatry has several weaknesses:

- The focus on individual risk factors found in psychiatric literature restricts the conceptual scope to psychiatric phenomena and ignores the fact that right-wing extremist orientations develop as a way to deal with social challenges. Although current approaches often propose multilevel models of radicalization that include social and societal factors, it remains unclear how individual and social factors interact in the formation of right-wing extremist orientations. Often, social aspects are conceptualized simply in terms of bad influence by peers or lack of social support that add to individual mental health risks.
- Despite little evidence for psychological causes of radicalization, psychiatric treatment is part of P/CVE programs such as the EU's Radicalization Awareness Network (Al-Attar, 2019). However, treatment recommendations often do not go beyond standard psychiatric treatment, such as medication and psychotherapy (RAN Practitioners., 2021a). That suggests that by treating mental health symptoms it is also possible to treat extremism. Moreover, the P/CVE approach also suggests that extremism is manageable through closer psychiatric screening and risk assessment, thus expanding the reach and tasks of psychiatric structures. The combination of psychiatric treatment of right-wing extremism and expansion of security policy tasks to psychiatry can be described as a form of top-down psychiatrization (Beeker et al., 2021). Top-down psychiatrization is a process driven by institutional and political agents in which an increasing number of people and areas of life become subject to psychiatric knowledge and practices.
- Difficulties and challenges for practitioners working with right-wing extremist patients have received little attention so far. Not only do right-wing extremist orientations conflict with ethical principles of the medical profession like, for example, treatment regardless of ethnicity, sexual orientation, or gender, but treatment of right-wing patients may also lead to tensions and conflicts with patients and staff who have experienced discrimination related to right-wing extremism.
- The conceptual focus in psychiatric literature lies almost exclusively on radicalization into violent extremism. As a result, radicalization processes that do not lead to violent acts do not receive sufficient attention.
- Often, right-wing extremism is subsumed under the label extremism, neglecting the specifics of right-wing extremism. Despite sharing some characteristics with, for example, Islamism, right-wing extremism develops in different social contexts and manifests in different ways (Bjørgo and Aasland, 2019).

To conclude, a research perspective is needed that can conceptually integrate psychological and social factors so that right-wing extremism becomes visible as a relationship persons form toward their social environment. Research should also be able to grasp the specifics of right-wing extremism in its violent as well as non-violent forms. Conceptually, a promising starting point can be found, for example, in the concept of orientation as it was developed within the German research on right-wing extremism among youth in the 1990's (Held et al., 1996; Marvakis, 1996, 2020). That approach understands right-wing extremism as an orientation aid for leading one's life in a complex and challenging social environment.

Since the trajectories into right-wing extremism are diverse, a qualitative methodology would be best suited to explore the interplay of individual, social, and societal aspects within the biography of right-wing extremists. If it is better understood how right-wing orientations develop during life course, it should be possible to work out if and how these orientations relate to psychiatric diagnoses and whether treatments are needed that take the specifics of right-wing orientations into account. The few studies that exist on therapeutic work with right-wing extremist patients understand right-wing extremism primarily as affinity toward violence (Ebrecht-Laermann et al., 2017; Hardtmann, 2017; Henkel et al., 2019). Instead, a broader scope that also includes right-wing extremism as an ideological orientation is needed. In this context, approaches from psychoanalytic social psychology, which emphasize the importance of far-right ideology for maintaining a psychological balance may be helpful (Busch et al., 2016; Lohl, 2021).

However, shifting the focus of research from psychological risk factors to processes of orientation in social contexts does not mean that psychiatry would not benefit from a better understanding of right-wing extremism. But instead of trying to cure right-wing extremism by psychiatric means, psychiatry needs to concentrate on the practical challenges of working with persons belonging to the far-right political spectrum. Research-based concepts and appropriately trained personnel are needed to deal professionally with these challenges. Understanding the limitations of psychiatry in this manner can potentially contribute to an effective and just use of resources in psychiatric institutions.

## Data Availability Statement

The original contributions presented in the study are included in the article, further inquiries can be directed to the corresponding author.

## Author Contributions

FS and PB developed the initial idea for the article, conducted literature search, and interpreted literature. FS wrote the first draft. All authors contributed to preparing the final version of the manuscript, approve the final version to be published, and agree to be accountable for all aspects of the work.

## Funding

We acknowledge funding by the MHB Open Access Publication Fund supported by the German Research Association (DFG).

## Conflict of Interest

The authors declare that the research was conducted in the absence of any commercial or financial relationships that could be construed as a potential conflict of interest.

## Publisher's Note

All claims expressed in this article are solely those of the authors and do not necessarily represent those of their affiliated organizations, or those of the publisher, the editors and the reviewers. Any product that may be evaluated in this article, or claim that may be made by its manufacturer, is not guaranteed or endorsed by the publisher.

## Footnotes

^1^As terminological discussions would go beyond the scope of the article, we chose to use the most well-known terms. However, it should be noted that there is considerable debate on the analytical value of terms like right-wing populism and extremism.
